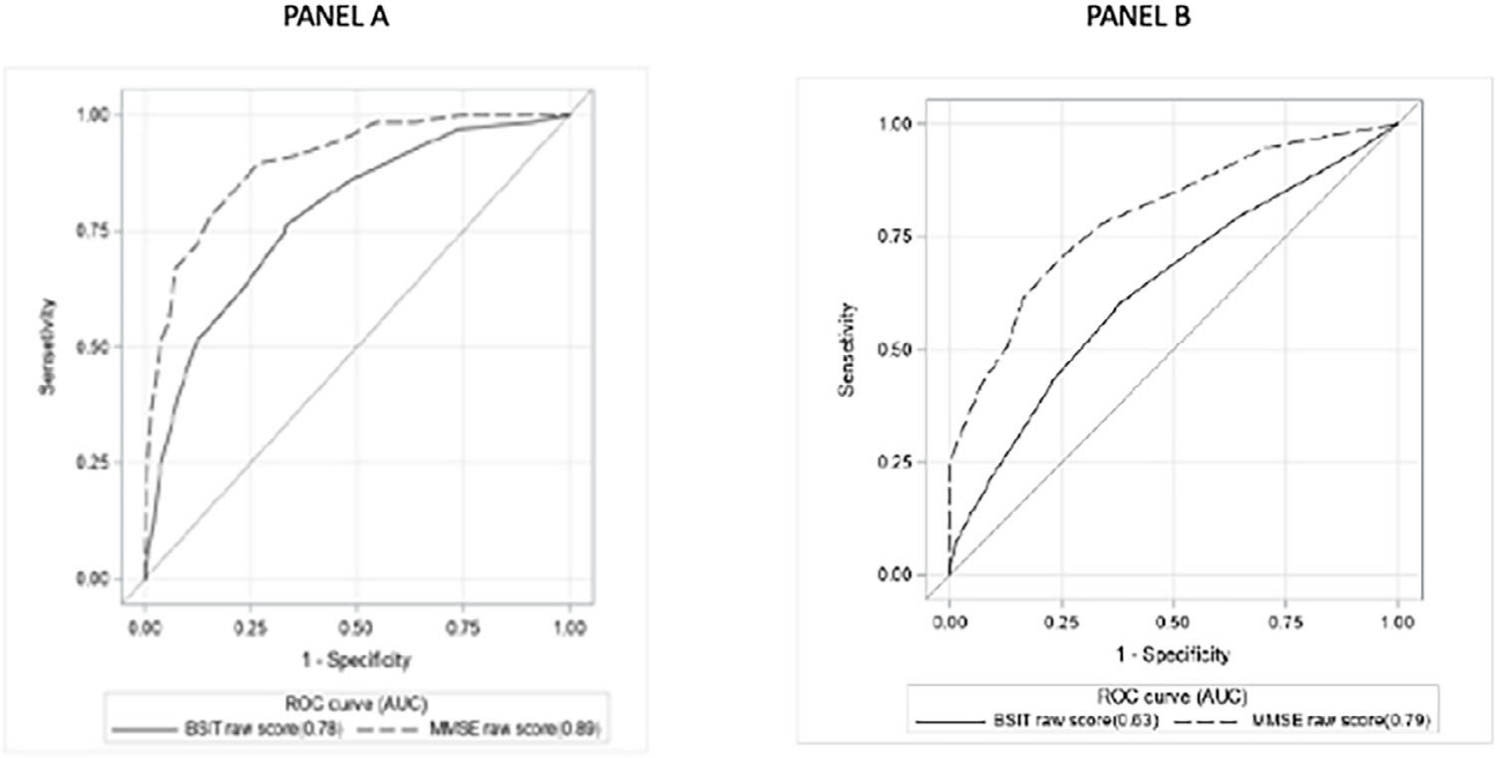# Performance of a smell identification test vs. the mini‐mental status exam for the detection of dementia and cognitive impairment among ethnically diverse persons with cognitive concerns in primary care

**DOI:** 10.1002/alz.092026

**Published:** 2025-01-09

**Authors:** Jose A. Luchsinger, Lenfis Valdez, Dahiana Rosario, Joseph P Eimicke, Stephanie A Silver, Jian X Kong, Jeanne Teresi, Terry E. Goldberg, Davangere Devanand

**Affiliations:** ^1^ Columbia University Irving Medical Center, New York, NY USA; ^2^ New York State Psychiatric Institute, New York, NY USA

## Abstract

**Background:**

Early detection of dementia and cognitive impairment is recommended for persons 65 years and older during wellness primary care visits. The importance of early detection has increased with the availability of new treatments for early Alzheimer's disease (AD). However, there is no clear approach for early detection in primary care. Odor identification deficits predict AD in epidemiological studies and may be useful for early detection. Our objective was to compare the accuracy of a short odor identification test with a short cognitive screening test for the detection of dementia and cognitive impairment in elderly persons with cognitive concerns.

**Method:**

This was a cross‐sectional study of 600 participants 65 years and older, without known mild cognitive impairment (MCI) or dementia, with cognitive concerns, attending primary care practices in New York City. The odor identification test was the Brief Smell Identification Test (BSIT). The comparator test was the Mini Mental Status Exam II (MMSE). Cognitive diagnoses were made using the National Alzheimer’s Coordinating Center Uniform Data set (NACC‐UDS) version 3 forms with slight modifications by a diagnosis consensus committee. Test performance was compared using Receiver Operating Characteristic analyses.

**Result:**

The mean age of the sample was 72.65 ± 6.31 years, 73.3% were female, 63.3% were Hispanic, 17.5% non‐Hispanic Black, and 27.0% non‐Hispanic White; 23.5% had normal cognition, 27.6% had cognitive impairment‐not mild cognitive impairment (MCI), 31.1% had amnestic MCI, 5.6% had non‐amnestic MCI, and 12% had dementia. The MMSE was superior to the BSIT in detecting dementia (AUC 0.89 vs 0.78, p =0.0007, Figure Panel A) and any cognitive impairment (AUC 0.79 vs 0.63, p <0.0001, Figure Panel B). Combining abnormal scores in the BSIT (< 9) to MMSE (< 24) improved the MMSE’s specificity (0.98 combined vs. 0.92 MMSE alone) and positive predictive value (PPV) in detecting cognitive impairment (0.98 combined vs. 0.95 MMSE alone).

**Conclusion:**

The MMSE was superior to the BSIT in detecting dementia and cognitive impairment in primary care but using both tests improved specificity and PPV for identifying persons with subjective complaints needing further cognitive and biomarker evaluation.